# Complete Genome Sequences of 12 B1 Cluster Mycobacteriophages, Gareth, JangoPhett, Kailash, MichaelPhcott, PhenghisKhan, Phleuron, Phergie, PhrankReynolds, PhrodoBaggins, Phunky, Vaticameos, and Virapocalypse

**DOI:** 10.1128/MRA.01387-18

**Published:** 2019-02-21

**Authors:** Kristofer A. Apellido, Divya Balchander, Matthew C. Erlich, Jakub K. Gocal, Wiktoria A. Gocal, Selam Haile, Annette K. Kang, Sravya Koduri, Maanasa Natrajan, Ayush A. Parikh, Ravi K. Tata, Tru A. Walor, Brianna M. Wong, Sprikena Nako, Trinh Tran, Eshraq Islam, Mirium P. Mammen, Drexel University SEA-PHAGES annotators 2016, Drexel University SEA-PHAGES annotators 2017, Drexel University SEA-PHAGES annotators 2018, Sarah L. Ball, David W. Bollivar, Kristen A. Butela, Welkin H. Pope, Matthew T. McDonald, Christine A. Tang, Ritu R. Dalia, Kevin P. W. Smith, Joy L. Little, Alison E. Moyer, Susan M. R. Gurney

**Affiliations:** a Department of Biology, Drexel University, Philadelphia, Pennsylvania, USA; b Center for Life Sciences Education, The Ohio State University, Columbus, Ohio, USA; c Department of Biological Sciences, Illinois Wesleyan University, Bloomington, Illinois, USA; d Division of Natural and Health Sciences, Seton Hill University, Greensburg, Pennsylvania, USA; e Department of Biological Sciences, University of Pittsburgh, Pittsburgh, Pennsylvania, USA; Queens College

## Abstract

Twelve B1 cluster mycobacteriophages were isolated from soil samples collected in Philadelphia, PA, USA, using Mycobacterium smegmatis mc^2^ 155 as a host, and were sequenced. The genome sequences range in size from 66,887 bp to 68,953 bp in length and have between 99 and 105 putative protein-coding genes.

## ANNOUNCEMENT

Mycobacteriophages are viruses that infect and replicate within mycobacteria. Based on their genome sequences, mycobacteriophages have been classified into 29 clusters. The B cluster is the second-most abundant cluster, with 286 members (phagesdb.org). This cluster has been subdivided into 8 subclusters (B1 to B8). In this paper we describe 12 B1 cluster mycobacteriophages isolated and annotated at Drexel University, Philadelphia, PA, USA.

All 12 bacteriophages were isolated from soil samples collected in Philadelphia, PA, USA. The bacterial host used was Mycobacterium smegmatis mc^2^ 155. Phage Gareth was isolated by adding 7H9 liquid medium to the soil sample. The liquid was filtered using a 0.22-µm filter and then incubated with Mycobacterium smegmatis mc^2^ 155 at 37°C for 48 h. The other 11 bacteriophages were isolated by adding 7H9 broth with glycerol, albumin dextrose (AD) supplement, CaCl_2_, and Mycobacterium smegmatis mc^2^ 155 to the soil samples. They were shaken at 220 rpm at 37°C for 96 h. The samples were centrifuged, and the supernatant was removed and filtered with a 0.22-µm filter. Plaque assays were performed to confirm the isolation of the bacteriophages. The bacteriophages were plaque-purified and amplified, then double-stranded DNA (dsDNA) was extracted using the Promega Wizard DNA cleanup kit. The genomes were individually sequenced with the Illumina MiSeq platform by the Pittsburgh Bacteriophage Institute. Sequencing libraries were prepared from genomic DNA with a NEB Ultra II kit or an Illumina TruSeq Nano kit. Forty-eight libraries were multiplexed and sequenced with an Illumina MiSeq platform, yielding at least 100,000 150-base single-end reads for each genome sequence, which was sufficient to provide at least 150-fold coverage (see Table 1). Raw reads were assembled with Newbler 2.9 with default settings, each yielding a single-phage contig, which was checked for completeness, accuracy, and phage genomic termini with Consed 29. After sequencing, the genomes were annotated with the following databases and software: DNA Master 5.23.2 (http://cobamide2.bio.pitt.edu), Glimmer 3.02 (1), GeneMark 2.5p (2), Starterator, Phamerator (https://phamerator.org) (3), PhagesDB BLAST (phagesdb.org/blast), NCBI BLASTP, the NCBI Conserved Domain Database (4, 5), HHPRED 3.0 beta (6), Aragorn 1.2.38 (7), tRNAscanSE 2.0 (8), and PECAAN (http://pecaan.kbrinsgd.org). The genomes were screened for repeated motifs with MEME software 5.0.2 (9).

**TABLE 1:** Twelve newly isolated B1 cluster mycobacteriophages

Phage name	GenBank accession no.	SRA accession no.	Length (bp)	G+C (%)	Protein-coding genes (no.)	Genome sequencing coverage (×)
Gareth	MH399775	SRR7774427	68,866	66.5	103	1,363
JangoPhett	MG757159	SRR7774424	68,673	66.5	102	230
Kailash	MF919511	SRR7774425	68,953	66.3	100	1,518
MichaelPhcott	MH576958	SRR7774426	68,494	66.4	100	1,299
PhenghisKhan	MG757164	SRR7774431	68,727	66.3	102	1,177
Phergie	MG757165	SRR7774432	68,716	66.3	103	1,276
Phleuron	MH316568	SRR7774429	68,527	66.4	100	962
PhrankReynolds	MH155874	SRR7774430	68,721	66.3	102	1,549
PhrodoBaggins	MH399786	SRR7774422	68,873	66.4	105	1,334
Phunky	MF919528	SRR7774423	68,375	66.5	99	1,100
Vaticameos	MH590588	SRR7774421	66,887	66.5	99	1,326
Virapocalypse	MF919539	SRR7774428	68,682	66.5	103	882

The genomic features of these bacteriophages are summarized in Table 1. The genome sequence sizes range from 66,887 to 68,953 bp and have a GC content from 66.3% to 66.5%. Similar to other B1 cluster bacteriophages (10), they contain ∼100 coding sequences (CDS) and no tRNA genes in any of the genomes.

Of these bacteriophage sequences, Phergie and PhenghisKhan are the most similar to each other, with 99% identity over 100% coverage (NCBI BLAST), and the most diverse are PhenghisKhan and Vaticameos, which share 98% identity over 93% coverage (NCBI BLAST). The genomic regions exhibiting the highest level of nucleotide diversity occurred between nucleotide positions 46 kb and 58 kb. Differences between the bacteriophage sequences include an HNH endonuclease gene (*gp10*) present in JangoPhett, Kailash, PhrodoBaggins, Phunky, and Virapocalypse, similar to that in phage Murdoc (GenBank accession number JN638752). Gareth and Vaticameos have a truncated version of the gene. Repeated motifs were identified; for example, one 50 nucleotide motif shared by bacteriophages Phunky, Gareth, Virapocalypse, JangoPhett, and Vaticameos was identified 42 nucleotides upstream of a shared forward gene, which has no known function.

### Data availability.

The genome sequences reported here have been deposited in DDBJ/ENA/GenBank under the accession numbers provided in Table 1 (BioProject number PRJNA488469). The SRA/DRA/ERA accession numbers are also provided in Table 1.
